# The Antioxidant and Anti-Inflammatory Properties of Rice Bran Phenolic Extracts

**DOI:** 10.3390/foods9060829

**Published:** 2020-06-24

**Authors:** Nancy Saji, Nidhish Francis, Lachlan J. Schwarz, Christopher L. Blanchard, Abishek B. Santhakumar

**Affiliations:** 1 Australian Research Council (ARC) Industrial Transformation Training Centre (ITTC) for Functional Grains, Graham Centre for Agricultural Innovation, Charles Sturt University, Wagga Wagga, NSW 2650, Australia; nsaji@csu.edu.au (N.S.); nfrancis@csu.edu.au (N.F.); lschwarz@csu.edu.au (L.J.S.); CBlanchard@csu.edu.au (C.L.B.); 2 School of Biomedical Sciences, Charles Sturt University, Locked Bag 588, Wagga Wagga, NSW 2678, Australia; 3 School of Animal and Veterinary Sciences, Charles Sturt University, Locked Bag 588, Wagga Wagga, NSW 2678, Australia; 4 School of Agricultural and Wine Sciences, Charles Sturt University, Locked Bag 588, Wagga Wagga, NSW 2678, Australia

**Keywords:** rice bran, polyphenols, oxidative stress, antioxidant, inflammation, anti-inflammatory

## Abstract

Oxidative stress and inflammation are known to be linked to the development of chronic inflammatory conditions, such as type 2 diabetes and cardiovascular disease. Dietary polyphenols have been demonstrated to contain potent bioactivity against specific inflammatory pathways. Rice bran (RB), a by-product generated during the rice milling process, is normally used in animal feed or discarded due to its rancidity. However, RB is known to be abundant in bioactive polyphenols including phenolic acids. This study investigates the antioxidant and anti-inflammatory effects of RB phenolic extracts (25, 50, 100, and 250 µg/mL) on RAW264.7 mouse macrophage cells stimulated with hydrogen peroxide and lipopolysaccharide. Biomarkers of oxidative stress and inflammation such as malondialdehyde (MDA), intracellular reactive oxygen species, nitric oxide and pro-inflammatory cytokines such as interleukin-6 (IL-6), monocyte chemoattractant protein 1 (MCP-1), interleukin-10 (IL-10), tumor necrosis factor-α (TNF-α), interleukin-12, p70 (IL-12p70), and interferon-γ (IFN-γ) were measured in vitro. Treatment with RB extracts significantly decreased the production of MDA, intracellular reactive oxygen species, nitric oxide and pro-inflammatory cytokines (IL-6, IL-12p70, and IFN-γ) when compared to the control. It is proposed that RB phenolic extracts, via their metal chelating properties and free radical scavenging activity, target pathways of oxidative stress and inflammation resulting in the alleviation of vascular inflammatory mediators.

## 1. Introduction

Oxidative stress is generated due to an imbalance between the endogenous antioxidant systems in our body and free radicals such as reactive oxygen species [1]. This consequently results in chronic inflammation leading to cardiovascular complications [2]. The key role of macrophages in the innate immune system is to engulf foreign agents, eliminate apoptotic cells and resolve inflammation via inflammatory cytokines and other mediators [3]. During an oxidative stress environment or a chronic inflammatory state, malondialdehyde (MDA), intracellular reactive oxygen species, nitric oxide, pro-inflammatory cytokines such as interleukin-6 (IL-6), monocyte chemoattractant protein 1 (MCP-1), interleukin-10 (IL-10), tumor necrosis factor-α (TNF-α), interleukin-12, p70 (IL-12p70), and interferon-γ (IFN-γ) are known to be secreted by activated macrophages [4,5].

MDA is generated as a result of the peroxidation between polyunsaturated fatty acids [6], as lipids are susceptible to oxidation due to their molecular structure which is abundant in reactive double bonds [7]. MDA is known to interlink with proteins (e.g., lysine to generate lysine–lysine cross-links) and subsequently result in interactions between oxidized low-density lipoprotein and macrophages, to promote the pathogenesis of cardiovascular disease [8]. Generally, cellular antioxidants such as catalase, superoxide dismutase and glutathione peroxidase are known to maintain the homeostatic levels of reactive oxygen species in the vasculature. However, the excess production of reactive oxygen species as a result of oxidative stress is recognized to result in the impairment of cell membrane, proteins, and nucleic acids leading to a series of vascular restructures resulting in atherosclerosis [1]. Nitric oxide, a gaseous lipophilic free radical cellular messenger, is generally known to protect against cardiovascular diseases by regulating blood pressure, impeding platelet aggregation and inhibiting smooth muscle cell proliferation. However, under oxidative stress conditions, nitric oxide is recognized to change from being a protective agent to being an infectious agent [9]. Moreover, during inflammation, activated macrophages are recognized to secrete several pro-inflammatory cytokines to further up-regulate the inflammatory responses. For example, IL-6 is a key cytokine involved in the synthesis and secretion of C-reactive protein which is one of the risk factors for the development of cardiovascular disease [10]. MCP-1 is involved in atherosclerotic plaque formation as it allows monocytes to pass from the lumen to the sub-endothelial space where they become foam cells, initiating fatty streak formation [11]. IL-10 is known to have a cardioprotective role as it is recognized to inhibit the adhesion of monocytes to endothelial cells and result in the destabilization of atherosclerotic plaque [12]. Similarly, TNF is normally recognized as a modulator of both cardiac contractility and peripheral resistance, but increased levels of TNF are known to result in cardiovascular complications [13]. In stimulated mouse macrophages, the increased production of IL-12p70 has been previously observed as a result of protein kinase C activation, which is known to control multiple physiological processes in the heart [14,15]. IFN-γ is a macrophage-activating factor, vital for both innate and adaptive immunity. It is produced by T cells and is known to initiate the generation of reactive oxygen species, and is therefore expressed at high levels during cardiovascular events such as atherosclerosis [16]. Therefore, the regulation of these inflammatory responses, by modulating the production of pro-inflammatory and oxidant markers has been the key metabolic target towards the treatment of vascular disorders.

Several synthetic drugs are currently available in the treatment of acute inflammatory states, however, their long-term use has been associated with gastrointestinal, cardiac and renal complications [17]. Moreover, a rise in health-conscious consumers has resulted in the search for functional foods with nutritional and disease-preventive properties [18]. Natural plant-derived phytochemicals, such as polyphenols and phenolic acids, have been demonstrated to target pathways of inflammation and oxidative stress [18]. Furthermore, it is believed that polyphenols, via their free radical scavenging, metal-chelating properties and blunting cellular signalling pathways, modulate the risk factors associated with chronic ailments including cardiovascular disease and cancer [19]. Phenolic compounds are known to exert anti-inflammatory activity through the regulation of cellular activities in inflammatory cells, modulating enzymes associated with the arachidonic acid metabolism and by blunting the release of pro-inflammatory molecules [19].

Rice is the seed of the grass species *Oryza sativa*, and has been cultivated around the globe for centuries and is considered a staple food. During rice milling, the rice bran (RB) layer is removed due to its rancidity associated with storage and is primarily used as animal feed. However, RB is known to be abundant in macronutrients and bioactive compounds such as *p*-coumaric acid, ferulic acid, and caffeic acid [20]. Although studies have demonstrated that RB phenolic compounds have the potential to exhibit antioxidant and/or anti-inflammatory properties [2], their impact on specific pathways of inflammation in an oxidative stress-induced/inflammatory environment has not been studied. The current study aimed to determine the antioxidant and anti-inflammatory effect of RB phenolic extracts on RAW264.7 mouse macrophage cells stimulated with hydrogen peroxide (H_2_O_2_) and lipopolysaccharide (LPS). Biomarkers of oxidative stress such as MDA, intracellular reactive oxygen species, nitric oxide and pro-inflammatory cytokines such as IL-6, MCP-1, IL-10, TNF-α, IL-12p70, and IFN-γ were measured in vitro.

## 2. Materials and Methods

### 2.1. Chemicals and Reagents

Unless otherwise stated, all the chemicals and reagents used in this study were acquired from Sigma-Aldrich (St Louis, MI, USA) and BD Biosciences (Franklin Lakes, NJ, USA).

### 2.2. Rice Bran Extract Preparation

Stabilization of RB was previously conducted by SunRice, Leeton, Australia using a drum-drying method. Extraction of the RB phenolic compounds was performed using an acetone/water/acetic acid (70:29.5:0.5, *v*/*v*) mixture, and characterized using ultra-high-performance liquid chromatography coupled to an 2,2’-Azino-bis 3-ethylbenzothiazoline-6-sulfonic acid-online system and mass spectrometry (Agilent Technologies) [20]. Prior to the cell culture studies, the extract was reconstituted in 50% dimethyl sulfoxide (DMSO).

### 2.3. Cell Culture Conditions

Experiments were conducted on RAW264.7 cells purchased from Sigma-Aldrich (St Louis, MO, USA). RAW264.7 cells were cultured in high-glucose Dulbecco’s modified Eagle’s medium (DMEM) (Sigma-Aldrich: D6429) supplemented with 10% fetal bovine serum and 1% 10,000 U/mL penicillin–10 mg/mL streptomycin at 37 °C in 5% CO_2_. Cultured RAW264.7 cells were used before reaching the 8th passage.

### 2.4. Cytotoxicity Assessment

A resazurin red cytotoxicity assay, as described by Saji, Francis [21], was utilized to examine the effect of RB phenolic extracts on RAW264.7 cells. Briefly, RAW264.7 cells were seeded into 96-well plates at a density of 50,000 cells/well and incubated overnight in the DMEM complete media. The cell count for the experimental seeding was achieved with a Muse^®^ Cell Analyzer from Luminex Corporation (Austin, TX, USA). RAW264.7 cells were then treated with 200 µL of DMEM complete media containing RB phenolic extract (25 µg/mL, 50 µg/mL, 100 µg/mL, 250 µg/mL, 500 µg/mL, 750 µg/mL and 1000 µg/mL) for 6 h. For the positive control, 5 mM concentration of H_2_O_2_ was used, and 0.5% DMSO served as the negative control. Subsequently, all the treatment wells were emptied before incubation with 200 µL of resazurin red solution for an additional 4 h. The absorbance at 570 and 600 nm was measured on a microplate reader (FLUOstar Omega microplate reader, BMG Labtech, Offenburg, Germany) against a resazurin red blank. The percentage of cell viability was calculated. Each treatment was measured in quintuplicate.

### 2.5. Antioxidant Effect of RB Phenolic Extract

#### 2.5.1. Malondialdehyde Determination

RAW264.7 cells (500,000 cells/well) were seeded into 6-well plates and incubated for 24 h. RB phenolic extracts at varying concentrations (25 µg/mL, 50 µg/mL, 100 µg/mL, 250 µg/mL) were then incubated with the cells for 6 h. To induce oxidative stress, RAW264.7 cells were subjected to 12 h incubation with a 500 µM concentration of H_2_O_2_. For MDA determination, a Lipid Peroxidation (MDA) Assay Kit (Sigma-Aldrich: MAK085) was used according to the manufacturer’s instructions. Briefly, the cell lysate was centrifuged for 10 min at 13,000× *g* to remove any insoluble material. Subsequently, 600 µL of freshly prepared thiobarbituric acid (TBA) solution was combined with 200 µL of the sample supernatant and the previously prepared MDA standards (0 nmol, 4 nmol, 8 nmol, 12 nmol, 16 nmol, and 20 nmol) to form the MDA–TBA adduct. All the samples were incubated at 95 °C for 1 h and subsequently placed in an ice bath for 10 min. The sensitivity of each sample was enhanced by adding 300 µL of 1-butanol, followed by centrifugation for 3 min at 16,000× *g* to separate the layers. The 1-butanol layer (the top layer) was then transferred to another tube and evaporated at 55 °C. The remaining residue was dissolved in 200 µL of water and transferred to a 96-well plate for analysis. Absorbance was measured at 532 nm using a microplate reader (FLUOstar Omega microplate reader, BMG Labtech, Offenburg, Germany). Each treatment was measured in quintuplicate.

#### 2.5.2. Intracellular Reactive Oxygen Species Generation

RAW264.7 cells were seeded at a density of 50,000 cells/well into a black, clear-bottom 96-well plate and incubated for 24 h. The cells were then incubated with RB extracts (25 µg/mL, 50 µg/mL, 100 µg/mL, 250 µg/mL) for 6 h. The DMSO-treated cells served as the control. Oxidative stress was induced by treatment with 500 µM concentration of H_2_O_2_ for 12 h. Formation of reactive oxygen species was determined by removing the old media and replenishing with 50 µM 2,7-dichlorofluorescein diacetate (Sigma-Aldrich: 35845) and incubating for an additional 30 min in the dark at 37 °C. The assessment of intracellular reactive oxygen species levels was conducted by measuring the fluorescence using a fluorescence microplate reader (FLUOstar Omega microplate reader, BMG Labtech, Offenburg, Germany) at 480 nm excitation and 530 nm emission at 37 °C. Each treatment was measured in octuplicate.

### 2.6. Anti-Inflammatory Effect of RB Phenolic Extract

#### 2.6.1. Nitric Oxide Determination

RAW264.7 cells at a density of 500,000 cells/well were seeded into a 6-well plate and incubated for 24 h, followed by treatment with 25 µg/mL, 50 µg/mL, 100 µg/mL, 250 µg/mL RB phenolic extracts for 6 h. DMSO-treated cells served as the control. To induce inflammation, RAW264.7 cells were further subjected to 24 h incubation with 1 µg/mL of LPS. The supernatant was collected by centrifugation (3000× *g*, 10 min, 4 °C). To assess the nitric oxide levels, the cell supernatants and standards (400 µL) were combined with 400 µL of Griess reagent (Sigma-Aldrich: G4410) and incubated for 15 min in the dark at room temperature. Sodium nitrite was used to generate a standard curve (10 μM, 20 μM, 40 μM, 60 μM, 80 μM and 100 μM). Absorbance was measured at 540 nm using a microplate reader (FLUOstar Omega microplate reader, BMG Labtech, Offenburg, Germany). Each treatment was measured in quintuplicate.

#### 2.6.2. Inflammatory Cytokine Determination

Seeding of RAW264.7 cells (500,000 cells/well) was conducted in a 6-well plate and incubated for 24 h. After confluency was reached, the RAW264.7 cells were treated with 25 µg/mL, 50 µg/mL, 100 µg/mL, 250 µg/mL RB phenolic extracts and DMSO (control) for 6 h. Inflammation was induced with the addition of LPS (1 µg/mL) and the cells were incubated for an additional 24 h, after which, the supernatant was collected by centrifugation (3000 × *g*, 10 min, 4 °C) and stored at −20 °C before analysis. The anti-inflammatory effect of RB phenolic extracts on inflammatory cytokines (IL-6, MCP-1, IL-10, TNF-α, IL-12p70, and IFN-γ) was determined using a BD™ Cytometric Bead Array Mouse Inflammation Kit (BD Biosciences: 552364). Briefly, 50 μL of the diluted samples (1:40) and standards were combined with 50 μL of the mixed capture beads and 50 μL of the mouse inflammation PE detection reagent. This was then incubated for 2 h in the dark at room temperature. After which, 1 mL of wash buffer was added to each assay tube and centrifuged (200 × *g*, 5 min). Following centrifugation, the supernatant was discarded, and the bead pellet was suspended in 300 μL of fresh wash buffer. Each sample was vortexed for 3–5 s and immediately inspected using a Gallios^TM^ Flow Cytometer (Beckman Coulter, CA, USA). Kaluza Flow Cytometry Analysis Software (Beckman Coulter, CA, USA) was used for conducting the data analysis. Each treatment was measured in quintuplicate.

### 2.7. Statistical Analysis

GraphPad Prism 7 software (GraphPad Software Inc., San Diego, California, USA) was utilized for statistical analysis by one-way analysis of variance (ANOVA), followed by post-hoc Tukey’s multiple comparisons test. Data were considered statistically significant when *p* < 0.05. Any significant statistical interactions were included in the analysis where applicable. The results are reported as mean ± standard deviation (SD).

## 3. Results

### 3.1. Cytotoxicity of RB Phenolic Extracts on RAW264.7 Cells

**Figure 1:**
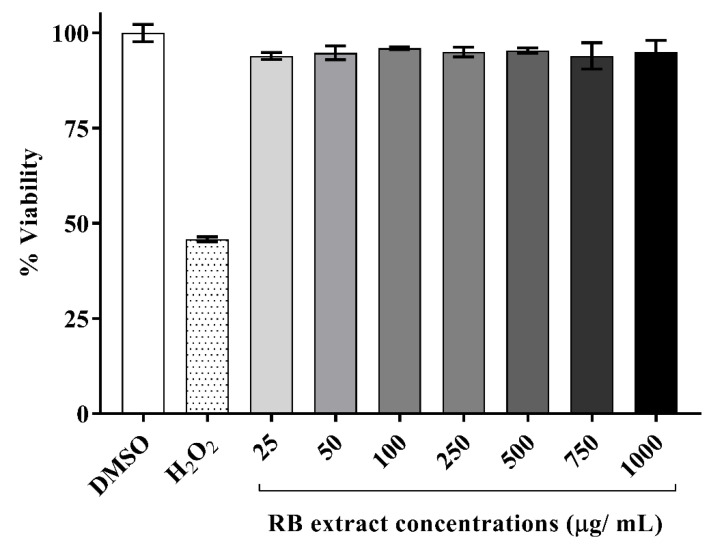
Cytotoxicity of RAW264.7 cells post-exposure to various doses of RB phenolic extracts (6 h treatment). The RB phenolic extracts did not display any cytotoxic effect on the RAW264.7 cells at the concentrations (25–1000 µg/mL) tested (*n* = 5). Data are presented as mean ± SD. Rice bran, RB.

A time-course study was conducted on a range of incubation periods varying from 2–24 h (data not shown) from which a 6 h incubation period was selected as the optimal time point associated with cell viability. It was observed that RAW264.7 cells, post-exposure to RB phenolic extracts for 6 h, did not display any toxic effects at any of the doses tested (25–1000 µg/mL) when compared to the DMSO control. Therefore, the optimal and physiologically attainable concentrations of RB phenolic extract selected for further examinations were between 25 and 250 µg/mL (Figure 1).

### 3.2. Antioxidant Properties of RB Phenolic Extract

#### 3.2.1. Malondialdehyde Concentration

**Figure 2:**
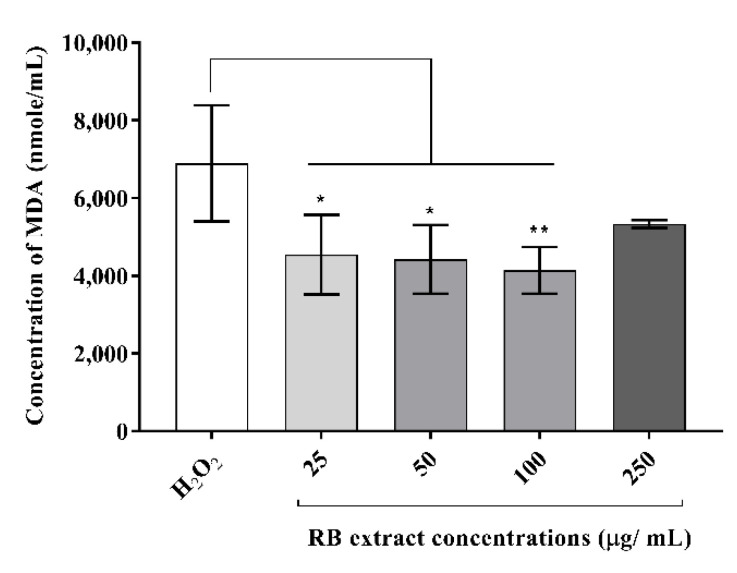
The impact of the RB phenolic extract treatment on oxidative stress-induced RAW264.7 macrophage cells. A significant reduction in the MDA concentration (nmol/mL) is observed after treatment with the different concentrations (25–100 µg/mL) of RB phenolic extracts compared to the control (*n* = 5). The level of significance is indicated by the asterisks, whereby * *p* < 0.05, ** *p* < 0.01. Data are presented as mean ± SD. Mouse macrophage cell, RAW264.7; hydrogen peroxide, H_2_O_2_; rice bran, RB.

MDA concentrations (nmol/mL) were significantly reduced in oxidative stress-induced RAW264.7 cells after treatment with 25 µg/mL (*p* < 0.05), 50 µg/mL (*p* < 0.05) and 100 µg/mL (*p* < 0.01) of RB phenolic extract compared to the H_2_O_2_ control (Figure 2). However, the treatment with 250 µg/mL RB phenolic extract did not reduce the MDA levels.

#### 3.2.2. Intracellular Reactive Oxygen Species Generation

**Figure 3:**
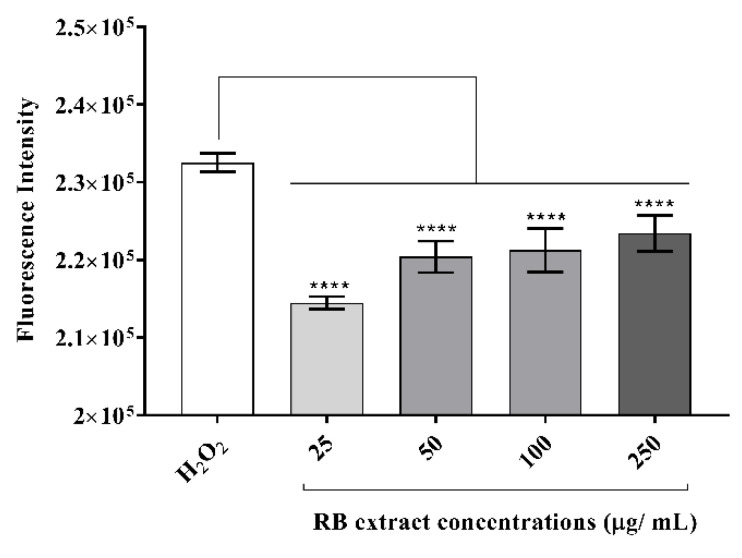
The effect of RB phenolic extracts on intracellular reactive oxygen species generation in oxidative stress-stimulated RAW264.7 cells. A significant reduction in intracellular reactive oxygen species generation, as indicated by the differences in the fluorescence intensity, is observed after treatment with the different concentrations (25–250 µg/mL) of RB phenolic extracts compared to the control *(n* = 8). The level of significance is indicated by the asterisks, whereby **** *p* < 0.0001. Data are presented as mean ± SD. Hydrogen peroxide, H_2_O_2_; rice bran, RB.

RAW264.7 cells induced with H_2_O_2_ showed a significant reduction in the generation of intracellular reactive oxygen species after treatment with 25–250 µg/mL (*p* < 0.0001) of RB phenolic extract when compared to the control (Figure 3).

### 3.3. Anti-Inflammatory Effect of RB Phenolic Extract

#### 3.3.1. Nitric Oxide Determination

**Figure 4:**
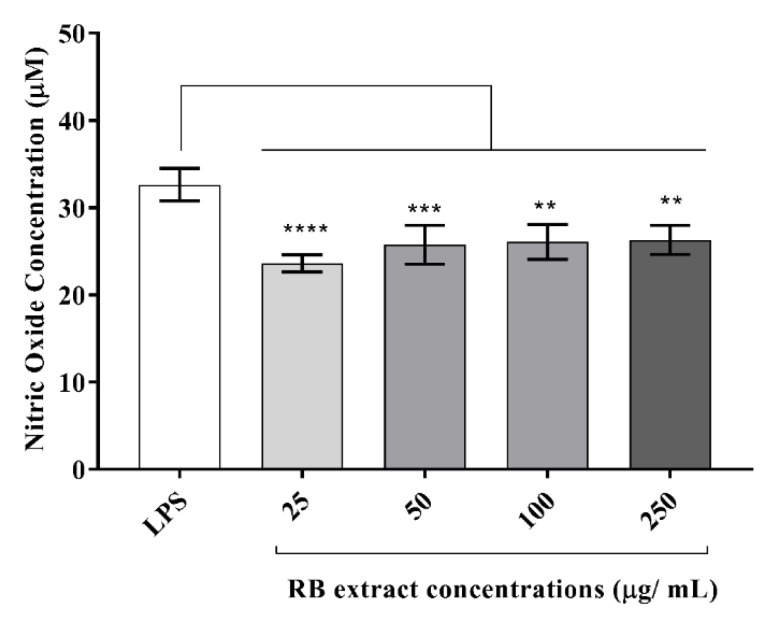
Effect of RB phenolic extract on nitric oxide levels in RAW264.7 cells induced with LPS. A significant reduction in the nitric oxide levels is observed after treatment with the different concentrations (25–250 µg/mL) of RB phenolic extracts compared to the control (*n* = 5). The level of significance is indicated by the asterisks, whereby ** *p* < 0.01, *** *p* < 0.001, **** *p* < 0.0001. Data are presented as mean ± SD. Lipopolysaccharide, LPS; rice bran, RB.

RB phenolic extracts at all concentrations (25–250 µg/mL) reduced the expression of nitric oxide levels in LPS-stimulated RAW264.7 cells (Figure 4).

#### 3.3.2. Inflammatory Cytokine Determination

**Figure 5:**
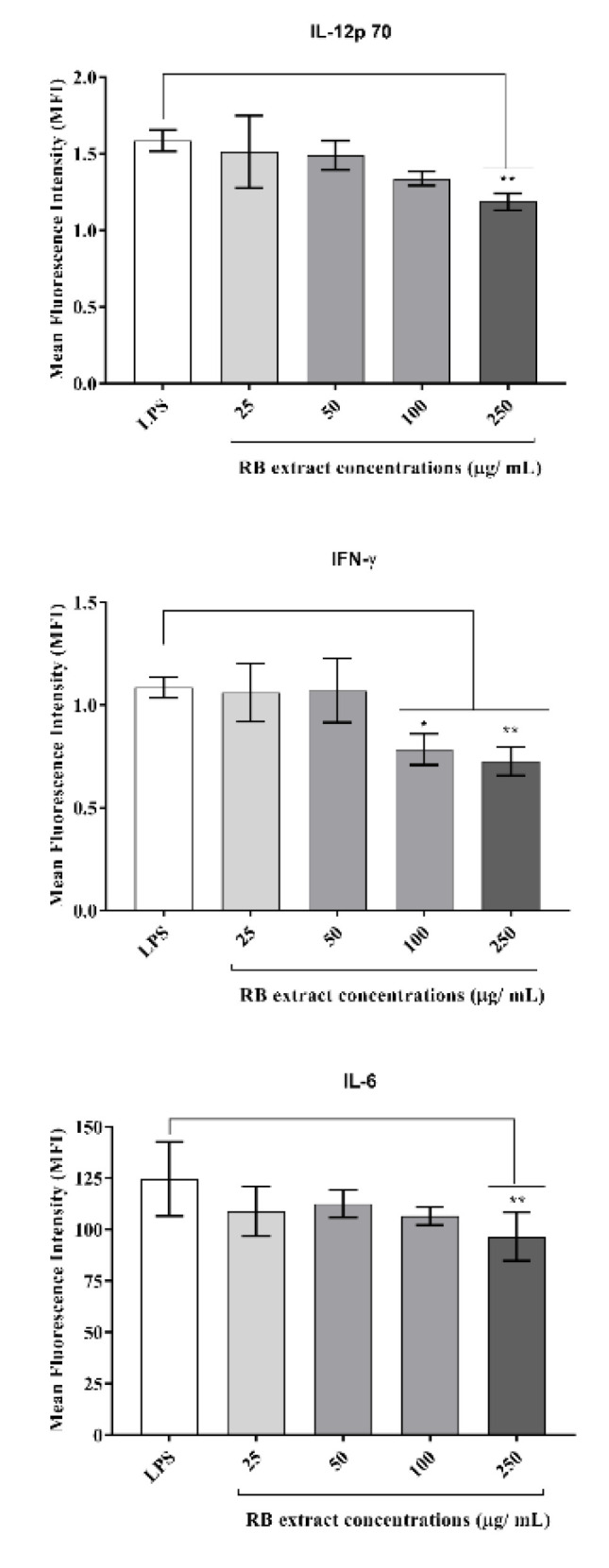
The effect of RB phenolic extracts in modulating pro-inflammatory cytokines. A significant reduction in IL-12p70 and IL-6 cytokines is observed after treatment with 250 µg/mL (*p* < 0.01). Furthermore, the pre-treatment with 100 µg/mL (*p* < 0.05) and 250 µg/mL (*p* < 0.01) of the RB phenolic extract is observed to have resulted in a significant reduction in the IFN-γ cytokine compared to the control (*n* = 5). The level of significance is indicated by the asterisks, whereby * *p* < 0.05, ** *p* < 0.01. Data are presented as mean ± SD. Lipopolysaccharide, LPS; rice bran, RB.

It was observed that treatment with 250 µg/mL (*p* < 0.01) of RB phenolic extract reduced the expression of IL-12p70 and IL-6 under a pro-inflammatory environment. Furthermore, the RB phenolic extract at 100 µg/mL (*p* < 0.05) and 250 µg/mL (*p* < 0.01) concentrations reduced IFN-γ compared to the LPS control (Figure 5). No significant differences in the regulation of the other cytokines tested were observed (data not shown for IL-10, MCP-1, and TNF-α).

## 4. Discussion

Both oxidative stress and inflammation are known to be precursor risk factors that play a central role in the development of chronic conditions such as cardiovascular disease [2]. Studies have proposed that polyphenols possess anti-inflammatory properties by virtue of their free radical scavenging, enzyme and cell signalling pathway modulating effects [19]. The current study has demonstrated that phenolic extracts present in RB have significant antioxidant and anti-inflammatory properties under oxidative stress-induced/inflammatory environment in macrophage cells. The drum-dried RB sample used in this study was previously characterized and some of the bioactive compounds identified in the extract were caffeic acid, ethyl vanillate, ferulic acid, feruloyl glycoside, *p*-coumaric acid, shikimic acid, sinapic acid, syringic acid, tricin and vanillic acid. Among which, ferulic acid and its isomers were identified to be the predominant compound in the extract with tricin having the most antioxidant activity. Furthermore, the examination of the total phenolic content and antioxidant activity revealed that the drum-dried RB extract had a total free phenolic content of 362.17 ± 34.16 GAE/100 g of RB with an antioxidant activity of 975.33 ± 20.24 Fe^2+^/100 g of RB and a total bound phenolic content of 160.65 ± 5.52 GAE/100 g of RB with an antioxidant activity of 551.91 ± 8.82 Fe^2+^/100 g of RB. It is believed that the antioxidant and anti-inflammatory effects observed in this study could be due to the synergistic action of the antioxidant-rich phenolic compounds identified in the extract [20].

An increase in free radicals results in the overproduction of MDA and reactive oxygen species levels in the body. Hence, they serve as an accurate biomarker for the detection of oxidative stress and overall antioxidant status [4]. In this study, RAW264.7 cells stimulated with H_2_O_2_ resulted in a significant reduction in MDA concentration (nmol/mL) after treatment with 25, 50 and 100 µg/mL of RB phenolic extract (Figure 2). Furthermore, it was observed that all RB extract concentrations (25–250 µg/mL) reduced the intracellular reactive oxygen species levels under a H_2_O_2-_stimulated oxidative stress environment (Figure 3). Other studies have also demonstrated that plant-derived bioactive compounds can alleviate free radical production in vitro under oxidative stress conditions [22,23,24]. Curcumin has been shown to decrease MDA and reactive oxygen species levels in macrophage cells that were induced with oxidative stress conditions. The authors have highlighted curcumin to have increased the activity of antioxidant enzymes and activated the Nrf2-Keap1 pathway [22]. In addition, pigeon pea extracts rich in cyanidin-3-monoglucoside were also observed to prevent the reduction of antioxidant enzyme activity and decrease MDA production in oxidative stress-induced macrophage cells [23]. By reducing hydrogen peroxidase, reactive oxygen species generation has been shown to increase free metal ion production, consequently producing highly reactive hydroxyl radicals. Polyphenols, due to their thermodynamically lower redox potential, reduce these oxidizing free radicals via their metal chelating ability [19]. The results observed in this study are a clear demonstration of the ability of RB polyphenols to reduce the highly oxidizing free radicals produced in an oxidative stress environment. In summary, it is believed that the antioxidant potential of polyphenols is correlated to their capacity to suppress reactive oxygen species formation, the inhibition of free radical-producing enzymes and/or the upregulation of antioxidant defences. The structure of functional groups in polyphenols also plays an important role in their antioxidant capacity. We believe that the synergistic action of polyphenols, including the phenolic acids present in the RB extracts by virtue of the mechanisms listed above, is blunting MDA and reactive oxygen species generation in an oxidative stress environment in vitro.

During inflammation, the activation of macrophage occurs as a result of a cascade of events that are mediated by the nuclear factor kappa-light-chain-enhancer of activated B cells (NF-κB) and mitogen-activated protein kinase (MAPK) pathways [25,26]. Macrophages stimulated with LPS result in the release of NF-κB protein, which acts as a transcription factor that promotes inflammation [25]. Similarly, activated MAPKs contribute to the phosphorylation of downstream targets, including protein kinases and transcription factors, which subsequently facilitates the transcription of MAPK-regulated genes. This process results in the production of mediators such as pro-inflammatory cytokines and nitric oxide in stimulated macrophage cells [26]. Since pro-inflammatory cytokines are recognized to play a vital role in cell signalling and systemic inflammation, the excessive production of pro-inflammatory cytokines may result in tissue destruction and the development of several pro-inflammatory processes [27].

In the present study, the stimulation of RAW264.7 cells with LPS was observed to up-regulate several inflammatory markers which were subsequently suppressed by pre-treatment with the RB phenolic extract. This included a significant decrease in the nitric oxide levels and pro-inflammatory cytokine (IL-6, IL-12p70, and IFN-γ) production in RAW264.7 cells stimulated with LPS (Figure 4 and Figure 5). However, no significant differences were observed in the IL-10, MCP-1, and the TNF-α production post-treatment with RB phenolic extracts (data not shown). Previous studies have also revealed that nitric oxide levels in LPS-induced RAW264.7 macrophages can be suppressed via treatment with extracts that have a high polyphenol content [28]. Rheosmin, a naturally occurring phenolic compound, was noted to dose-dependently suppress nitric oxide production by inhibiting the activity of NF-κB [29]. Moreover, it is also believed that nitric oxide inhibition by phenolic compounds may be achieved as a result of the substitution of the hydroxyl functional group [28]. Polyphenols are known to favourably modulate the activity of arachidonic acid metabolizing enzymes such as cyclooxygenase (COX), lipoxygenase (LOX) and nitric oxide synthase (NOS) [19]. The observed effect on the alleviation of the nitric oxide levels could be a direct impact of the synergistic action of RB polyphenols in inhibiting the key inflammation-mediating enzymes.

The results observed in this study demonstrate that RB phenolic compounds effectively down-regulated the expression of IL-6, IL-12p70, and IFN-γ production in LPS-stimulated RAW264.7 cells. IL-12 is a heterodimeric interleukin that plays an important role in defence against intracellular pathogens [30]. It is characterized by the high-level secretion of both IFN-γ and TNF-α [30]. IL-12p70 is one of the first pro-inflammatory cytokines released after antigens from the foreign agents, apparent in the microenvironment, and the secretion of IL-12p70 cytokine is critical for the initiation and polarization of the appropriate immune response [31]. IFN-γ is a cytokine that is critical for both innate and adaptive immunity and is widely recognized for its pro-inflammatory capability against infectious agents [32]. Furthermore, it is predominantly produced by natural killer cells and is known to inhibit viral replication directly [33]. An essential mediator of the inflammatory signalling pathway induced by LPS is a transcription factor known as a signal transducer and an activator of transcription 3 (STAT3) [34]. Activated STAT3 is recognized to translocate to the nucleus and regulate the transcription of inflammation-related genes. Upon LPS stimulation, the activation of STAT3 through the IL-6 signalling pathway can be observed to result in increased IL-6 production [34]. Polyphenols have been known to modulate the aforementioned pro-inflammatory products by inhibiting the enzymes associated with pro-inflammatory effects (COX-2, LOX and NOS); MAPK, protein kinase-C and activated protein-1 activation; and NFĸB inhibition [19]. It is believed that RB polyphenols, by virtue of their structure-activity relationship and free radical scavenging attributes, might potentially target the mechanisms detailed above. Further studies are warranted to understand the role of RB phenolic extracts on specific molecular mechanisms associated with inflammation.

In the body, phytochemicals are processed as xenobiotics. This is because the human body is only capable of distinguishing between nutrients and constituents that are not nutrients, and is unable to differentiate between beneficial, neutral and toxic compounds. However, many of these phytochemicals are known to activate the adaptive cellular response pathways to oxidative stress by acting as low-dose stressors or pro-oxidants [35]. In the present study, treatment with lower doses of the RB phenolic extract (25 µg/mL) was observed to have resulted in a more pronounced inhibitory effect to certain markers compared to the higher doses (Figure 2, Figure 3 and Figure 4). This occurs through a phenomenon known as hormesis, a biphasic dose-response with stimulation in low-doses and inhibition in higher doses (lesser activity) of the phytochemicals present in the RB extracts [36]. Furthermore, it is believed that the oversaturation of phenolic compounds, though excreted via the portal circulation in vivo, could result in the unfavourable activation of cellular signalling molecules . In addition, bioavailability is another factor that needs to be considered, as the effect of any dietary compound is mainly influenced by its bioavailability, or the proportion of the substance that actually enters the circulation when introduced into the body. Due to the remarkable diversity in the human population influenced by genetics and/or medications, the appropriate bioavailable dosage may be significantly different [35]. Furthermore, future studies that compare the in vitro effect of crude RB phenolic extracts against antioxidant/anti-inflammatory synthetic/therapeutic agents are warranted. This would also help provide novel insights into potential mechanisms associated with the mode of action of natural polyphenols in the pro-inflammatory process. Although the current study highlights the antioxidant and anti-inflammatory properties of RB at physiologically attainable concentrations in vitro, it is important to demonstrate similar effects in vivo due to the potential variation in the bioavailability of polyphenol sub-classes and individual predisposition. Well controlled human dietary intervention trials are warranted to confirm the thus observed in vitro impact of RB phenolic compounds.

## 5. Conclusions

This study highlights that RB phenolic extracts play a significant role in modulating the biomarkers of oxidative stress and inflammation in an oxidative stress/inflammatory environment in vitro. It is believed that the RB phenolic compounds, via their synergistic action, target pathways of inflammation and oxidative stress through their free radical scavenging potential, and modulate pro-inflammatory cytokine expression. The outcomes of this study suggest that RB phenolic extracts may potentially be used as a functional food alternative and could have preventive or therapeutic implications in chronic oxidative stress and inflammatory conditions. Further in vivo investigations that evaluate the bioactivity of RB phenolic extracts in pro-inflammatory populations are warranted.

## Abbreviations

ANOVA	Analysis of variance
COX	Cyclooxygenase
DMSO	Dimethyl sulfoxide
DMEM	Dulbecco’s modified Eagle’s medium
H_2_O_2_	Hydrogen peroxide
IFN-γ	Interferon-γ
IL-10	Interleukin-10
IL-12p70	Interleukin-12, p70
IL-6	Interleukin-6
LPS	Lipopolysaccharide
LOX	Lipoxygenase
MDA	Malondialdehyde
MAPK	Mitogen-activated protein kinase
MCP-1	Monocyte chemoattractant protein 1
NOS	Nitric oxide synthase
NF-κB	Nuclear factor kappa-light-chain-enhancer of activated B cells
RB	Rice bran
STAT3	Signal transducer and activator of transcription 3
SD	Standard deviation
TBA	Thiobarbituric acid
TNF-α	Tumor necrosis factor-α
